# How repetitive are genomes?

**DOI:** 10.1186/1471-2105-7-541

**Published:** 2006-12-22

**Authors:** Bernhard Haubold, Thomas Wiehe

**Affiliations:** 1 Department of Biotechnology & Bioinformatics, University of Applied Sciences Weihenstephan, Freising, Germany; 2 Institute of Genetics, Universität zu Köln, Cologne, Germany

## Abstract

**Background:**

Genome sequences vary strongly in their repetitiveness and the causes for this are still debated. Here we propose a novel measure of genome repetitiveness, the index of repetitiveness, *I*_r_, which can be computed in time proportional to the length of the sequences analyzed. We apply it to 336 genomes from all three domains of life.

**Results:**

The expected value of *I*_r _is zero for random sequences of any G/C content and greater than zero for sequences with excess repeats. We find that the *I*_r _of archaea is significantly smaller than that of eubacteria, which in turn is smaller than that of eukaryotes. Mouse chromosomes have a significantly higher *I*_r _than human chromosomes and within each genome the Y chromosome is most repetitive. A sliding window analysis reveals that the human *HOXA *cluster and two surrounding genes are characterized by local minima in *I*_r_. A program for calculating the *I*_r _is freely available at .

**Conclusion:**

The general measure of DNA repetitiveness proposed in this paper can be efficiently computed on a genomic scale. This reveals a broad spectrum of repetitiveness among diverse genomes which agrees qualitatively with previous studies of repeat content. A sliding window analysis helps to analyze the intragenomic distribution of repeats.

## Background

Repeat sequences are a common feature of prokaryote and eukaryote genomes [1-3] and in both types of organisms the selective neutrality or otherwise of extra copies of sequences has been debated for decades [3]. Since the start of the genomics era in the mid-1990s the hitherto unexpectedly large amount of repetitive sequences found in bacteria, which may account for more than 10% of the total genome, prompted a flurry of investigations of the functional and evolutionary significance of these elements [4]. More recently, Aras *et al*. surveyed 51 bacterial genomes to quantify the effect repeat sequences might have on genome plasticity due to intragenomic recombination [5]. The authors conclude that in bacteria repeats might be selected for their positive effect on the adaptability of their host [5]. In another *in silico *survey of 58 completely sequenced bacteria, Achaz *et al*. noted that inverted repeats are underrepresented in bacterial genomes due to their destabilizing effect on genome structure [6].

In eukaryotes the discrepancy between DNA content and apparent organismic complexity had been noted even before the discovery of the double helix leading to the conclusion that "The relationship between DNA and the size or number of genes is obscure" [[7], p. 462]. In the 1960s DNA reannealing studies uncovered that eukaryotic genomes contain a highly variable fraction of repetitive DNA. Since the sequencing of complex genomes these observations have been made precise: approximately 50% of the human genome is made up of repetitive sequences [8]. However, the term "repetitive sequences" encompasses a rather heterogeneous set of elements: 45% of the human genome is covered by transposons, 3% are repeats of less than a hundred base pairs (microsatellites and minisatellites), and 5% consist of recent duplications of large segments of DNA. Broadly similar observations have been made in other mammalian genomes [9-11]. The human genome contains low, but appreciable, genetic variation caused by transposable elements, indicating that transposable elements have been active over the short time span since humans diverged from their last common ancestor [12]. However, the decline of transposon activity in the hominoid lineage contrasts with more recent insertions in mouse, where new spontaneous mutations are 60 times more likely to be caused by transposition than in human [9].

The hypothesis that transposable elements are molecular parasites was originally designed to explain the apparently excessive DNA baggage of eukaryotes [13,14]. A number of contemporary observations support this view. Transposon-derived sequences are rare close to transcription start sites and inside coding regions, suggesting that insertions are usually deleterious [15]. Moreover, the four human *HOX *clusters and other highly regulated genomic regions contain very few transposable elements [8]. Direct deletion of megabase-sized regions devoid of known genes also seems to have no effect on mice, even though these regions contain elements that have been conserved since the emergence of mammals [16]. There is no contradiction between these observations and the fact that occasionally transposable elements can give rise to beneficial structures including novel gene regulatory regions [15] and the V(D)J recombination mechanism that generates the antibody diversity expressed by vertebrate B cells [17].

Since the publication of whole genome data, the quantification and classification of repeat elements has become a major area of research in computational biology [18,19]. Perhaps the best-known program for the detection of repeat elements is repeatmasker [20], which looks for two things: (1) tandem repeats of a few nucleotides, and (2) homology to known repetitive elements. This approach has the advantage of dealing with elements of known origin. Its disadvantage is that the presence of hitherto unknown repetitive elements might be missed. The program repeatfinder implements a highly efficient and more generic approach based on suffix trees that makes no assumptions about the type of repeat present [19]. Such methods can be used to compute, for example, the percentage of a given DNA sequence covered by repeats and most methods provide a means of checking the statistical significance of the repeats returned. Suffix trees allow the efficient detection of all exact repeats in a sequence. In contrast, the widely used relative simplicity factor (RSF) [21] is based on the local density of repeat motifs up to four bases long compared to their density in a shuffled version of the input sequence [22]. Application of the RSF to diverse genomes revealed that eukaryotes are characterized by an elevated "micro-repetitiveness" compared to prokaryotes [23].

What is lacking, though, is an all-inclusive measure of repetitiveness. Under the RSF repetitiveness is defined as a quantity that is minimized by shuffling the investigated sequence. As suggested by the term *simplicity *factor, studies of repetitiveness are related to investigations of complexity [24] – if repetitiveness is high, complexity is low, though the converse is not always true. For example, the "linguistic complexity" of a string *S *is defined as the number of substrings of lengths 2, 3, ..., |*S*| observed in *S *compared to the maximum number of substrings of these lengths [25]. A random DNA sequence with G/C content 0.5 has maximal complexity and minimal repetitiveness. However, a random DNA sequence with a G/C content of, say, 0.1 does not have maximal complexity, while its repetitiveness should still be minimal.

In this paper we propose a novel measure of repetitiveness which considers repeats of any length, takes into account G/C content, and does not necessitate shuffling for its computation. As explained in detail in the Methods Section, our index of repetitiveness, *I*_r_, is expected to be zero in random DNA sequences of any G/C content and length, and can be computed in time proportional to sequence length. We apply the *I*_r _to 303 sequenced bacterial genomes, 27 archaebacteria, and six model eukaryotes: baker's yeast (*Saccharomyces cerevisiae*), nematode worm (*Caenorhabditis elegans*), thale cress (*Arabidopsis thaliana*), fruit fly (*Drosophila melanogaster*), mouse (*Mus musculus*), and human (*Homo sapiens*).

## Results

**Figure 1:**
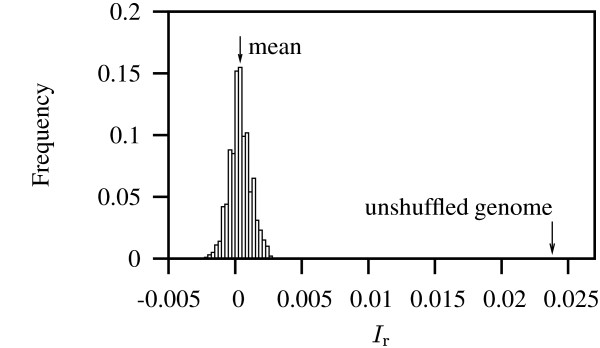
**The null distribution of *I*_r_**. The genome of bacteriophage λ was shuffled 1000 times and the *I*_r _computed; mean = 0.0004, sd = 0.0008.

Our first goal was to establish the null distribution of *I*_r_. This can be obtained by shuffling a genomic sequence. As an example we repeatedly randomized the genome of bacteriophage λ, which consists of 48,502 bp of DNA, and calculated the *I*_r _from these "repeatless" sequences. Figure 1 shows the resulting histogram, which is symmetrically distributed around a mean close to the expected zero (mean = 0.0004, sd = 0.0008). Further analysis of this distribution using the Shapiro-Wilk test [26] revealed that deviation from normality increased as more replicates were added (not shown). The reason for this was an increase in kurtosis (2.972 in Figure 1), while the skewness (0.078 in Figure 1) decreased with higher replication. Notice also that the *I*_r _of the unshuffled λ genome is significantly greater than its randomized version. This is not surprising, as biological sequences are no more random sequences of residues than prose is a random sequence of letters.

### Survey of *I*_r _values

**Figure 2:**
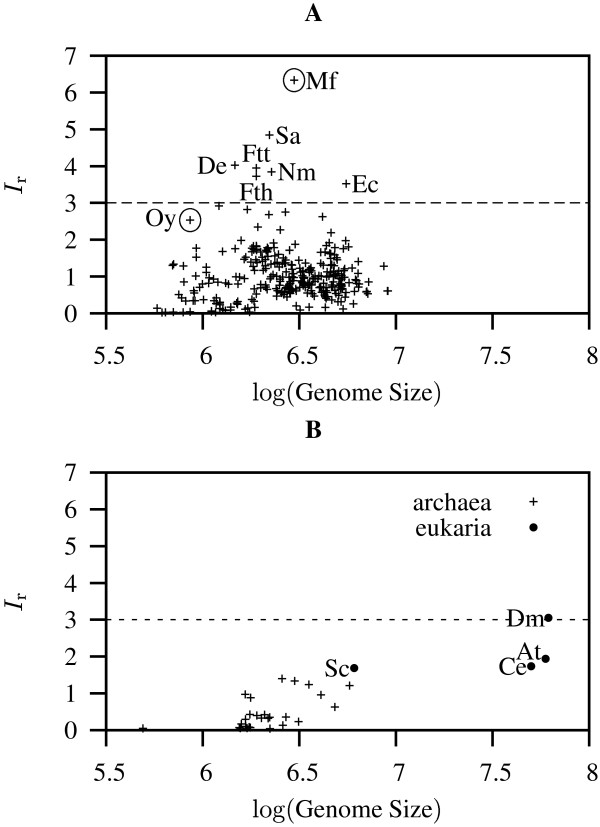
***I*_r _values of 334 completely sequenced genomes taken from the three domains of life**. *I*_r _values shown as a function of their log genome size; dashed lines delineate organisms with *I*_r _> 3. **A**: Eubacteria, circled values correspond to the genomes subjected to sliding window analysis in Figure 3; Mf: *Methylobacillus flagellatus *KT; Sa: *Streptococcus agalactiae *NEM316; De: *Dehalococcoides ethenogenes *195; Ftt: *Francisella tularensis *subsp. tularensis SCHU S4; Nm: *Neisseria meningitidis *MC58; Fth: *Francisella tularensis *subsp. holarctica; Ec: *Escherichia coli *O157:H7 EDL933; Oy: Onion yellows phytoplasma OY-M. **B**: archaebacteria and eukaryotes; Sc: *Saccaromyces cerevisiae; *Ce: *Caenorhabditis elegans*; At: *Arabidopsis thaliana*; Dm: *Drosophila melanogaster*.

We calculated *I*_r _values for 330 completely sequenced prokaryote genomes, as well as for representative eukaryotic model organisms: baker's yeast (*Saccharomyces cerevisiae*; 12 Mb surveyed), nematode worm (*Caenorhabditis elegans*; 100 Mb surveyed), thale cress (*Arabidopsis thaliana*; 119 Mb surveyed), and fruit fly (*Drosophila melanogaster*; 123 Mb surveyed). Figure 2A displays the *I*_r _values of eubacteria as a function of the log genome size [see Additional file 1 for a complete listing of prokaryote results]. In this domain of life *I*_r _was not correlated with log genome size (Pearson correlation = 0.046; *P *= 0.425). The average *I*_r _of eubacteria was 1.048. 94.7% of bacteria had an *I*_r _≤ 2. On the other hand, there were 7 bacteria where *I*_r _> 3, with the highest value found in *Methylobacillus flagellatus *KT (6.337; Figure 2A). The other members of this group were *Streptococcus agalactiae *NEM316 (*I*_r _= 4.842), *Dehalococcoides ethenogenes *195 (4.026), *Francisella tularensis *subsp. tularensis SCHU S4 (3.950), *Neisseria meningitidis *MC58 (3.842), *Francisella tularensis *subsp. holarctica (3.723), and *Escherichia coli *O157:H7 EDL933 (3.521; Figure 2A).

At the other extreme of the distribution, *Buchnera aphidicola *str. Bp had the smallest *I*_r _value (0.019), which was even smaller than that observed in phage λ (*I*_r _= 0.024; Figure 1). With one exception the ten eubacteria with the lowest *I*_r _values comprised only intracellular organisms sampled form the genera *Buchnera, Chlamydophila, Candidatus, Neorickettsia*, and *Rickettsia*. The exception was the highly abundant photosynthetic bacterium *Prochlorococcus marinus *subsp. marinus str. CCMP1375 [see Additional file 1].

Figure 2B displays the *I*_r _values of archaebacteria and eukaryotes. In archaebacteria *I*_r _was significantly correlated with log genome size (Pearson correlation = 0.562; *P *= 0.002), while in eukaryotes the correlation was not significant (Pearson correlation = 0.485; *P *= 0.515). The average *I*_r _of archaebacteria was 0.467, which is significantly smaller than that of eubacteria (Wilcoxon test, *P *= 3.15 × 10^-6^). The average *I*_r _of eukaryotes was 2.103, which is in turn significantly greater than either that of eubacteria (*P *= 4.3 × 10^-3^) or archaebacteria (*P *= 6.36 × 10^-5^). Among eukaryotes only *Drosophila melanogaster *had an *I*_r _> 3.

**Figure 3:**
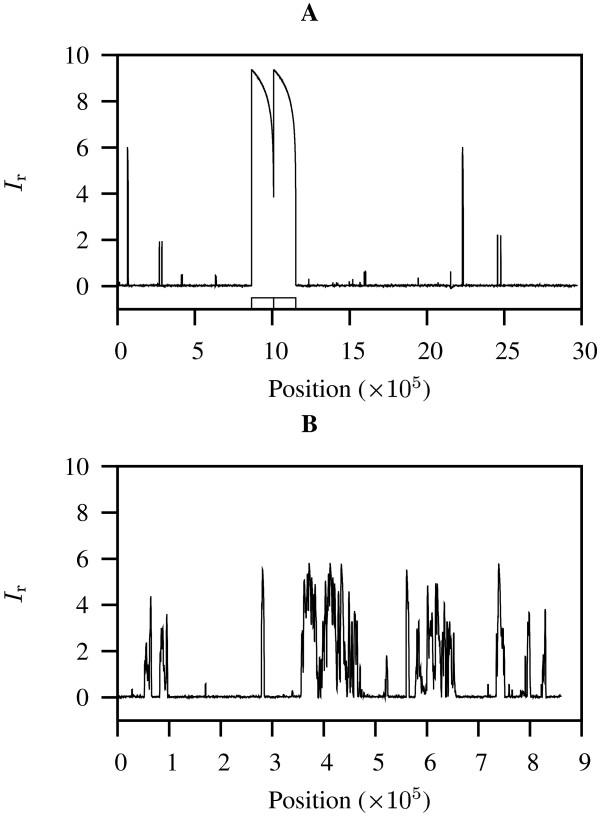
**Sliding window analyses of two bacterial genomes**. **A**: *Methylobacillus flagellatus *KT with tandem repeat comprising 143 kb (boxes); **B**: Onion yellows phytoplasma OY-M. The global *I*_r_-values of these two bacteria are circled in Figure 2A.

In order to further investigate some of the extreme *I*_r _values observed in eubacteria (Figure 2A), we subjected them to sliding window analyses. Figure 3A shows such an analysis for *M. flagellatus *KT and reveals that its global *I*_r _value (Figure 2A, Mf) was caused by two large peaks of local *I*_r _indicating the presence of a very long exact repeat (Figure 3A). This turned out to be a tandem repeat comprising an astonishing 143,034 bp. Removal of one copy of this duplication lead to a much deflated *I*_r _of 0.657. However, not all large *I*_r _values among eubacteria were caused by single exact repeats. Figure 3B displays a sliding window analysis of the genome of Onion yellows phytoplasma OY-M, which had a global *I*_r _value of 2.348 (Figure 2A, Oy). A scan of its local *I*_r _values indicated the presence of numerous regions of significant repetitiveness (Figure 3B).

The bacterium with the second highest global *I*_r_-value, *Strepotococcus agalactiae *NEM316 (*I*_r _= 4.842; Figure 2A) was an outlier among the other 14 streptococci investigated, which have an average *I*_r _of 1.665 [see Additional file 1]. Window analysis of *S. agalactiae *NEM316 revealed three exact repeats of 47 kb (not shown) and their removal resulted in an *I*_r _of 1.756. Similarly, *Escherichia coli *OH157:H7 EDL933 had an exceptionally high *I*_r _of 3.521 (Figure 2A) compared to the other five strains of *E. coli *sampled (average *I*_r_: 1.049; cf. Additional file 1). In this case window analysis of *E. coli *OH157:H7 EDL933 (not shown) highlighted a repeat region of approximately 100 kb located at positions 1,050,000–1,150,000 and 1,450,000–1,550,000, which contained several long exact repeats with the longest spanning over 41 kb. Removal of one copy of the 100 kb repeat region reduced the *I*_r _to 1.756.

### Mouse and human chromosomes

**Figure 4:**
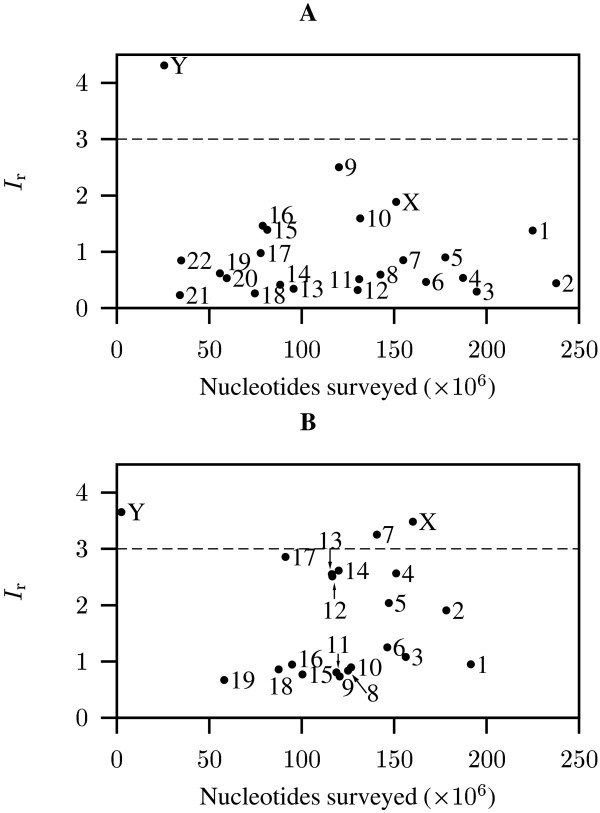
***I*_r _values as a function of the number of nucleotides surveyed in human (A) and mouse (B) chromosomes**. Dashed line delineates chromosomes with *I*_r _> 3.

The average *I*_r _for human chromosomes was 0.985 and values for individual chromosomes ranged from 0.229 in chromosome 21 to 4.313 in the Y chromosome (Figure 4A). The Y chromosome was the only human chromosome with *I*_r _> 3, which agrees with the view that it has the highest DNA turnover in the genome [8].

The average *I*_r _for mouse chromosomes was 1.773 (Figure 4B), which is significantly larger than that of humans (Wilcoxon test, *P *= 1.4 × 10^-3^). This agrees with the observation that the rodent lineage has experienced a higher rate of retro-transposition than hominoids [9]. Individual mouse chromosomes had *I*_r _values ranging from 0.7 in chromosome 19 to 3.654 in the Y chromosome. As in the human genome, the Y chromosome from mouse was characterized by the largest *I*_r_. In addition, chromosomes 7 and X had *I*_r _values > 3 (Figure 2B).

### HOX genes in human and D. melanogaster

**Figure 5:**
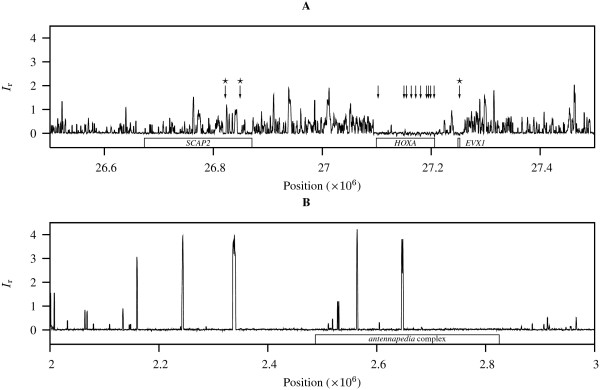
**Sliding window analysis of *HOX genes***. **A**: 1 Mb of human chromosome 7 containing the *HOXA *cluster. Arrows indicate runs of *I*_r _≤ 0 longer than 2 kb; starred arrows point to regions outside of the *HOXA *cluster, which consists of 13 individual genes. **B**: 1 Mb of chromosome 3R from *D. melanogaster *containing the antennapedia complex.

The *HOX *genes encode transcription factors that function as fundamental developmental switches in all animals. In human the four clusters of *HOX *genes contain very few insertion sequences [8]. To assess the effect of this on the landscape of human *I*_r _values, we carried out a sliding window analysis of 1 Mb around the *HOXA *cluster on chromosome 7. Figure 5A displays the conspicuous footprint of low *I*_r _values that coincides with the location of the *HOXA *cluster. In order to make this eye-ball analysis more quantitative, we searched the fragment of chromosome 7 displayed in Figure 5A for runs of *I*_r _≤ 0 that extended for at least 2 kb. This uncovered 13 intervals ranging in size from 2.1 to 4.1 kb (arrows in Figure 5). Ten of these intervals were located within the *HOXA *cluster. The remaining three arrows are marked by stars in Figure 5. Two of the corresponding regions with low *I*_r _values intersected with *SCAP2*, a *src *family associated phosphoprotein involved in signal transduction leading to T cell activation [27]. The last region of low *I*_r _outside of the *HOXA *region intersected with *EVX1*. This is a homologue of the even-skipped homeobox gene originally discovered in *D. melanogaster*. In vertebrates it is involved in eye development [28]. Human *EVX1 *is located just 42.73 kb upstream from the most 5' of the *HOXA *genes, *HOXA13 *(Figure 5).

A sliding window analysis of the *antennapedia *complex in *D. melanogaster*, which is homologous to part of the human *HOXA *cluster, revealed a very different topology of repetitiveness (Figure 5B). On a background of *I*_r _≈ 0, large peaks marked the presence of long exact repeats and the *antennapedia *cluster was not characterized by a conspicuous change in *I*_r _values.

## Discussion

"At this point we do not know what most of the DNA in eukaryotes is doing" [[29], p. 253]. Today, thirty-five years later, the function of apparently excess DNA in both eukaryotes and prokaryotes remains a topic of intense research activity [3]. Our method to quantify this excess DNA, the index of repetitiveness, is close in spirit to the investigation of linguistic complexity based on suffix trees [25]. Linguistic complexity is maximized in random sequences with equiprobable residues. Deviations from equiprobability lead to a reduction in complexity even if the sequence remains completely random. In contrast, in this paper we were interested in quantifying repetitiveness with respect to genome composition and to make this measure comparable across genomes. Our starting point was an investigation of the complement of repeats, the unique sequences. These are trivially easy to find, for example a sequence is always unique with respect to itself, and for this reason we have concentrated on *shortest *unique substrings. A shortest unique substring occurs only once in its parent string and cannot be reduced in length without losing its uniqueness. A genome with many long repeats contains many excessively long shortest unique substrings, while its shuffled version contains only the shortest unique substrings expected to be there by chance alone (cf. Methods). Since we have derived the latter quantity analytically [30], the *I*_r _is constructed as the logarithm of the ratio between the observed and expected aggregate number of nucleotides found in shortest unique substrings. At the cost of ignoring homology relationships, this measure has the advantage that it can be computed for any double-stranded DNA sequence and its expectation is always zero. It is also possible to estimate an *I*_r _value for sequences over alphabets other than the four nucleotides. In this case the quantity *A*_e _defined in Equation (2) can be estimated by shuffling the input sequence. For example, the *I*_r _of this paper is approximately 0.7.

Since the construction of the underlying suffix tree takes only time proportional to the length of the sequence analyzed, the *I*_r _can be computed in time proportional to the length of the input sequence. In contrast, traditional repeat analysis such as implemented in the program repeatmasker [20] runs in time proportional to the product of the length of query and subject sequence.

Like most suffix tree implementations, the suffix tree on which our analysis is based, is kept entirely in the main memory (RAM) of the computer [31]. This has the advantage of being relatively easy to implement. The disadvantage of this approach is that the amount of sequence data that can be analyzed in a single run of the program is limited by the available RAM rather than by the much cheaper hard disk space. We are currently studying advances in disk-based suffix tree construction [32] in order to break through the RAM barrier.

It may come as a surprise that the *I*_r _values for human and mouse chromosomes were within the range of *I*_r _values observed for less complex eubacterial genomes (Figure 2). However, this does not contradict the well-known fact that mammalian genomes are full of interspersed repeats, while bacteria usually contain fewer of these elements. The apparent paradox is due to the fact that the effect of interspersed repeats on the excess amount of exact repeats in a given genome – which is what the *I*_r _measures – depends not only on the fraction of sequence covered by repetitive elements; equally important is the number of mutations accumulated since the divergence of an interspersed repeat from its most recent ancestor. As a result of the mutation process, ancient repetitive elements may not contain longer motifs repeated elsewhere than the rest of the genome. The presence of such elements would leave the *I*_r _unchanged compared to the identical genome without them.

A similar argument applies to the interpretation of the high *I*_r _values found in the Y chromosomes of human and mouse. The two factors determining the accumulation of sequence polymorphisms, time to the most recent common ancestor and mutation rate, cannot be separated. In addition, the effective mutation rate differs between autosomes and the Y chromosome. Under neutrality the number of SNPs expected for a pair of homologous sequences is *θ *= 4*N*_e*μ*_, where *N*_e _is the effective population size and *μ *the rate of mutation. Since the effective population size of mammalian Y chromosomes is only one quarter that of autosomes, repeat pairs on the Y chromosome are broken up more slowly by mutations than elsewhere in the genome contributing to higher *I*_r _values.

It should be noted at this point that neither the mouse nor the human genome are completely sequenced to date. If new sequence data comes predominantly from regions that are difficult to sequence due to their repetitiveness, future editions of the human and mouse genomes are expected to have higher *I*_r_.

The *I*_r _values found in our whole genome analyses (Figure 2) correlate well with the relative simplicity factors (RSFs) reported previously [23] (Pearson correlation = 0.552, *P *= 3.3 × 10^-4^). This correlation is not perfect due to the fact that the RSF measures the local excess of short repeats, while the *I*_r _measures the excess of all repeats throughout the sequence. Moreover, no significant correlation between archaebacterial genome size and RSF was observed by Hancock [23], in contrast to our finding. This effect, however, is simply due to differences in sampling; if we reduce our sample of 27 archaebacterial genomes to the nine investigated by Hancock, the correlation between *I*_r _and log genome size also vanishes. In contrast, a tenfold increase in the number of bacterial genomes investigated between Hancock's and our study only confirmed the earlier diagnosis of no correlation between RSF and genome size.

The average *I*_r _for eubacteria was 1.048. However, it is clear that there are a few extreme *I*_r _values that inflate this average (Figure 2A). The largest *I*_r _for bacteria (or for any other organism) was found in *Methylobacillus flagellatus *KT (6.337). This value was the most extreme of a set of seven organisms with *I*_r _> 3 that also included the human pathogens *Neisseria meningitidis *MC58 and *Escherichia coli *O157:H7 EDL933 (Figure 2). In a previous survey of 58 bacteria, *Neisseria meningitidis *was already singled out as having a highly repetitive genome [6]. The low *I*_r _values found by us among obligately host-associated bacteria also agree with a known lack of repeats in these genomes [6]. While other bacteria appear to harbor repeats to increase genome plasticity [5], we speculate that intracellular symbionts and pathogens are less dependent on genome shuffling for their survival as they live in more stable environments. Our sliding window analyses revealed that the computation of *I*_r _values for entire genomes averages out sharp regional fluctuations in *I*_r _(Figures 3 and 5). In bacteria a high *I*_r _value may be caused by a few extreme duplications, as was the case for *M. flagellatus *KT (Figure 3A) and *S. agalactiae *NEM316. In the human genome the 13 genes making up the *HOXA *cluster were characterized by a 100 kb footprint of low *I*_r _values (Figure 5A). The fact that additional runs of low *I*_r _outside the *HOXA *cluster also coincided with known genes leads us to currently search the entire human genome for further regions of low *I*_r_.

## Conclusion

Investigations of repetitiveness are traditionally carried out using some form of alignment algorithm. Such algorithms tend to run in time proportional to the product of the length of the query and subject sequence. In this paper we present an approach that runs in time linear in the length of the input sequence. It is based on a comparison between the observed and expected sums of the lengths of shortest unique substrings. We apply the resulting index of repetitiveness, *I*_r_, to prokaryote and eukaryote genomes. Our global repetitiveness measures agree qualitatively with current knowledge about genome structure. However, a more detailed picture emerges by subjecting the genomes to window analyses. In the human genome the highly regulated *HOXA *cluster is known to lack insertion sequences. Accordingly, it is characterized by a footprint of low *I*_r_. This suggests that in mammalian genomes regions of low *I*_r _may be due to strong selection against mutagenesis by insertion sequences. If this is the case, scanning mammalian genomes for further intervals of low *I*_r _may reveal tracts under strong purifying selection.

## Methods

### Measuring repetitiveness

**Figure 6:**
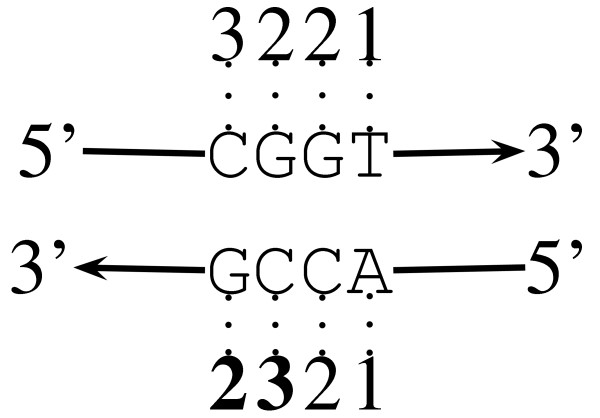
**Shortest unique substring lengths for the DNA sequence CGGT and its complement**. Starting from, say, the first nucleotide, three steps in the 3' direction are necessary to generate a unique substring. The numbers in bold correspond to suffix length plus one; see text for details.

In the following we derive a generic measure of repetitiveness in DNA sequences, the index of repetitiveness, *I*_r_. Consider a genome, *S*, consisting on its forward and reverse strands of 2*l *nucleotides. At each position *i *along this genome we can determine the length of the shortest unique substring starting at that position, *x*_*i*_. Such a shortest unique substring has the property that the substring *S *[*i*..*i *+ *x*_*i *_- 1] is unique, while *S *[*i*..*i *+ *x*_*i *_- 2] is not. Figure 6 shows the example sequence *S *= CGGT and the lengths of all the corresponding shortest unique substrings. Notice that no shortest unique substrings start at the two most 3' positions of the reverse strand. In that case we assign suffix length plus one as the shortest unique substring length (bold numbers in Figure 6). In other words, we pretend that each string is terminated by a unique "sentinel" character.

**Figure 7:**
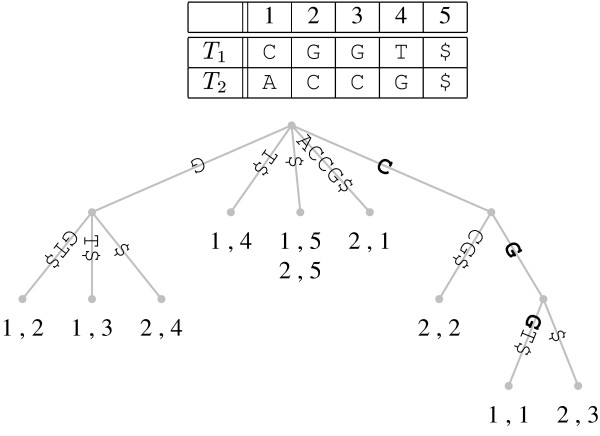
**Suffix tree corresponding to the forward and reverse strands of the example sequence CGGT (cf. Figure 6)**. Leaf labels consist of a string identifier, followed by the starting position of the suffix read from the root to the leaf. For example, the suffix GGT$ starts at position 2 in string #1. Any string starting at the root of the tree and ending on a terminal branch, e.g. the substring CGG shown in bold, is unique. CGG is also *shortest *unique because it extends only for one character on the external branch.

We have used suffix trees [31] to detect shortest unique substrings in genomic sequences. Figure 7 shows the suffix tree that corresponds to our example sequence. This tree is read as follows: the concatenated labels along a path leading from the root at the top to a leaf yield a suffix of the input string starting at the position indicated by the label of the leaf. Suffix trees have the useful property that any string starting at the root and ending somewhere on an internal branch is a repeated substring. For example, substring CG occurs at position 1 in *T*_1 _and at position 3 in *T*_2 _(Figure 7). Conversely, a string starting at the root and ending anywhere on an external branch, e.g. CGG, is a unique substring (cf. bold edge labels in Figure 7). Given a suffix tree, it is therefore easy to locate the *shortest *unique substrings starting at any position *i *in the genome by looking up the length of the path label from the root to the parent of the leaf referring to position *i*. This length is known as the *string depth *of a node, *s*. The desired length of the shortest unique substring starting at *i *is then simply *x*_*i *_= *s *+ 1.

**Figure 8:**
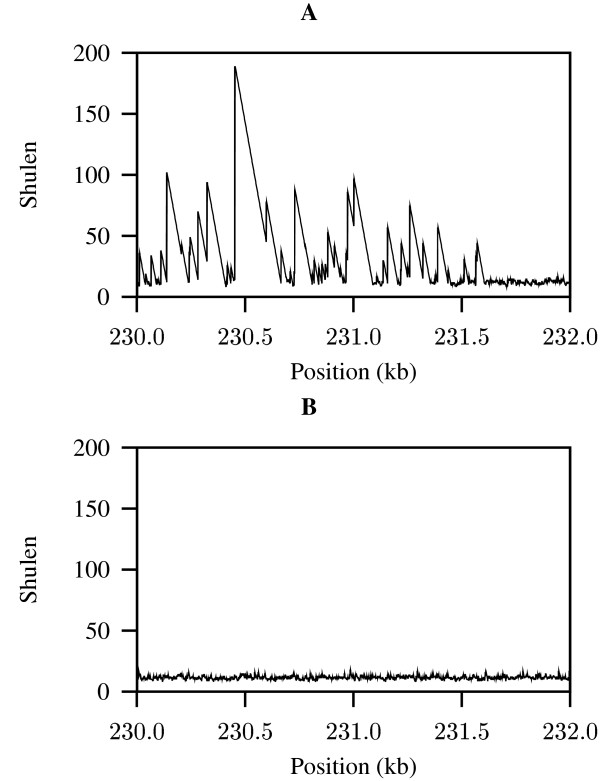
**Lengths of shortest unique substrings (shulen) along 2 kb of the genome of human pathogen *Mycoplasma genitalium***. **A**: Original sequence; **B**: shuffled sequence.

Figure 8A shows the value of *x*_*i *_along 2 kb of genomic sequence from the human pathogen *Mycoplasma genitalium*. The spikes in this curve correspond to unusually long shortest unique substrings, which are caused by repeats that are longer than expected by chance alone. We define the observed aggregate length of shortest unique substrings as

Ao=∑i=12lxi.     (1)
 MathType@MTEF@5@5@+=feaafiart1ev1aaatCvAUfKttLearuWrP9MDH5MBPbIqV92AaeXatLxBI9gBaebbnrfifHhDYfgasaacH8akY=wiFfYdH8Gipec8Eeeu0xXdbba9frFj0=OqFfea0dXdd9vqai=hGuQ8kuc9pgc9s8qqaq=dirpe0xb9q8qiLsFr0=vr0=vr0dc8meaabaqaciaacaGaaeqabaqabeGadaaakeaacqWGbbqqdaWgaaWcbaGaee4Ba8gabeaakiabg2da9maaqahabaGaemiEaG3aaSbaaSqaaiabdMgaPbqabaaabaGaemyAaKMaeyypa0JaeGymaedabaGaeGOmaiJaemiBaWganiabggHiLdGccqGGUaGlcaWLjaGaaCzcamaabmaabaGaeGymaedacaGLOaGaayzkaaaaaa@3FDE@

The quantity *A*_o _corresponds to the area under the curve shown in Figure 8A.

We have previously derived an exact expression for the number of shortest unique substrings of length *x *expected in a completely shuffled genome of a given length and G/C content, *N*_*x *_[30]. It is therefore convenient to define the expected aggregate length of shortest unique substrings as

Ae=∑xxNx.     (2)
 MathType@MTEF@5@5@+=feaafiart1ev1aaatCvAUfKttLearuWrP9MDH5MBPbIqV92AaeXatLxBI9gBaebbnrfifHhDYfgasaacH8akY=wiFfYdH8Gipec8Eeeu0xXdbba9frFj0=OqFfea0dXdd9vqai=hGuQ8kuc9pgc9s8qqaq=dirpe0xb9q8qiLsFr0=vr0=vr0dc8meaabaqaciaacaGaaeqabaqabeGadaaakeaacqWGbbqqdaWgaaWcbaGaeeyzaugabeaakiabg2da9maaqafabaGaemiEaGNaemOta40aaSbaaSqaaiabdIha4bqabaaabaGaemiEaGhabeqdcqGHris5aOGaeiOla4IaaCzcaiaaxMaadaqadaqaaiabikdaYaGaayjkaiaawMcaaaaa@3CC5@

Figure 8B shows the length of shortest unique substrings at each position along a shuffled version of the 2 kb fragment from the genome of *M. genitalium*. Notice that all the spikes indicating long repeats contained in the original sequence data (Figure 8A) have vanished, leaving a narrow baseline of shortest unique substring lengths. The quantity *A*_e _is the expectation of the area under this baseline curve.

The index of repetitiveness, *I*_r_, is now defined as the logarithm of the ratio of the observed aggregate shortest unique substring length and its theoretical expectation:

Ir=log⁡(AoAe).     (3)
 MathType@MTEF@5@5@+=feaafiart1ev1aaatCvAUfKttLearuWrP9MDH5MBPbIqV92AaeXatLxBI9gBaebbnrfifHhDYfgasaacH8akY=wiFfYdH8Gipec8Eeeu0xXdbba9frFj0=OqFfea0dXdd9vqai=hGuQ8kuc9pgc9s8qqaq=dirpe0xb9q8qiLsFr0=vr0=vr0dc8meaabaqaciaacaGaaeqabaqabeGadaaakeaacqWGjbqsdaWgaaWcbaGaeeOCaihabeaakiabg2da9iGbcYgaSjabc+gaVjabcEgaNnaabmaabaWaaSaaaeaacqWGbbqqdaWgaaWcbaGaee4Ba8gabeaaaOqaaiabdgeabnaaBaaaleaacqqGLbqzaeqaaaaaaOGaayjkaiaawMcaaiabc6caUiaaxMaacaWLjaWaaeWaaeaacqaIZaWmaiaawIcacaGLPaaaaaa@4002@

For genomes devoid of excess repeat sequences *I*_r _≈ 0, while for sequences with an excess of repeats *I*_r _> 0. We have written the program ir for calculating *I*_r_. The software is accessible using any standard web browser [33].

### Sequence data

All 330 completely sequenced prokaryote genomes contained in RefSeq [34] at the time of analysis were downloaded from the NCBI ftp-site (). Their accession numbers and *I*_r _values are provided in Additional file 1. Table 1 summarizes the sources of the six eukaryotic genomes analyzed in this study.

**Table 1:** The sources of the eukaryotic genomes analyzed in this study.

Organism	Source	Version
*A. thaliana*		n/a
*C. elegans*		ce2
*D. melanogaster*		dm2
*H. sapiens*		38
*M. musculus*		mm8
*S. cerevisiae*		SacCer1

### *I*_r _calculations and statistical analysis

All *I*_r _values presented in Figure 2 were computed from the complete genome data available. Unsequenced regions marked by Ns were removed to prevent artificial inflation of *I*_r_. The human and mouse genomes were too large for complete analysis with the computing equipment available to us. We therefore analyzed only individual chromosomes (Figure 4). With the exception of human and mouse chromosomes 1 and 2, all sequences were analyzed on their reverse and forward strands. Due to their sizes, only the forward strands of human and mouse chromosomes 1 and 2 were included in the computation of *I*_r_.

For the sliding window analyses (Figures 3 and 5) *A*_o _is computed as the sum of shortest unique substring lengths starting inside an interval of 1000 bp. Similarly, *A*_e _is a function of the local G/C content and window length (1000 in our case). The window is then moved by a tenth of its length, i.e. 100 bp, and the *I*_r _is recomputed.

The significance of differences between average values computed from sets of *I*_r _values was tested using the two-sample Wilcoxon test as implemented in the statistics software R [35].

## Availability and requirements

We have implemented *I*_r _computations in the program ir, which can be accessed via a web-interface at



The C source code of a stand-alone version of the program is also freely available from this web site under the terms of the GNU General Public License.

## Authors' contributions

BH designed and implemented the software, performed data analysis and contributed to the writing of the manuscript. TW initiated the study of shortest unique substrings, derived the null distribution of their lengths, and contributed to the writing of the manuscript. Both authors read and approved the final manuscript.

## Supplementary Material

Additional File 1 Supplementary Material. *I*_r _values for 330 completely sequenced prokaryote genomes sorted by *I*_r _or organism. Click here for file
