# Supplementary material for: How repetitive are genomes?
Source: BMC Bioinformatics. 2006 Dec 22;7:541. doi: 10.1186/1471-2105-7-541 (PMC1769404; doi:10.1186/1471-2105-7-541)
Supplement: Additional File 1 — Supplementary Material. Ir values for 330 completely sequenced prokaryote genomes sorted by Ir or organism. [file 1471-2105-7-541-S1.pdf]

Supplementary material for *How repetitive are genomes?*

Bernhard Haubold & Thomas Wiehe

July 4, 2006

Table 1 lists I_r values for 330 completely sequenced prokaryote genomes sorted by I_r and Table 2 list the same information sorted by organism.

Table 1: I_r values for prokaryote genomes sorted by I_r

No.	Accession	Organism	Size	I_r
1	NC_007947	Methylobacillus flagellatus KT	2971517	6.3367
2	NC_004368	Streptococcus agalactiae NEM316	2211485	4.8417
3	NC_002936	Dehalococcoides ethenogenes 195	1469720	4.0257
4	NC_006570	Francisella tularensis subsp. tularensis SCHU S4	1892819	3.9503
5	NC_003112	Neisseria meningitidis MC58	2272360	3.8422
6	NC_007880	Francisella tularensis subsp. holarctica	1895994	3.7227
7	NC_002655	Escherichia coli O157:H7 EDL933	5528445	3.5209
8	NC_005364	Mycoplasma mycoides subsp. mycoides SC str. PG1	1211703	2.9206
9	NC_002940	Haemophilus ducreyi 35000HP	1698955	2.8218
10	NC_002488	Xylella fastidiosa 9a5c	2679306	2.7542
11	NC_007575	Thiomicrospira denitrificans ATCC 33889	2201561	2.6811
12	NC_007712	Sodalis glossinidius str. 'morsitans'	4171146	2.6269
13	NC_005303	Onion yellows phytoplasma OY-M	860631	2.5371
14	NC_005956	Bartonella henselae str. Houston-1	1931047	2.3482
15	NC_004556	Xylella fastidiosa Temecula1	2519802	2.2695
16	NC_004337	Shigella flexneri 2a str. 301	4607203	2.1937
17	NC_005955	Bartonella quintana str. Toulouse	1581384	1.9798
18	NC_002695	Escherichia coli O157:H7	5498450	1.9753
19	NC_007613	Shigella boydii Sb227	4519823	1.9191
20	NC_006349	Burkholderia mallei ATCC 23344	2325379	1.9111
21	NC_003212	Listeria innocua Clip11262	3011208	1.8736
22	NC_007606	Shigella dysenteriae Sd197	4369232	1.8411
23	NC_003485	Streptococcus pyogenes MGAS8232	1895017	1.8374
24	NC_000907	Haemophilus influenzae Rd KW20	1830138	1.8248
25	NC_006086	Streptococcus pyogenes MGAS10394	1899877	1.8149
26	NC_005126	Photorhabdus luminescens subsp. laumondii TTO1	5688987	1.8043
27	NC_007384	Shigella sonnei Ss046	4825265	1.7863
28	NC_004116	Streptococcus agalactiae 2603V/R	2160267	1.7840
29	NC_002950	Porphyromonas gingivalis W83	2343476	1.7821
30	NC_004551	Tropheryma whipplei TW08/27	925938	1.7737
31	NC_006449	Streptococcus thermophilus CNRZ1066	1796226	1.7684
32	NC_002927	Bordetella bronchiseptica RB50	5339179	1.7593
33	NC_007797	Anaplasma phagocytophilum HZ	1471282	1.7544
34	NC_003116	Neisseria meningitidis Z2491	2184406	1.7507
35	NC_004606	Streptococcus pyogenes SSI-1	1894275	1.7407
36	NC_007296	Streptococcus pyogenes MGAS6180	1897573	1.7299
37	NC_007297	Streptococcus pyogenes MGAS5005	1838554	1.7275
38	NC_004757	Nitrosomonas europaea ATCC 19718	2812094	1.7172
39	NC_004070	Streptococcus pyogenes MGAS315	1900521	1.7159

Continued on next page

Prokaryote I_r sorted by I_r, continued from previous page				
No.	Accession	Organism	Size	I_r
40	NC_002745	Staphylococcus aureus subsp. aureus N315	2814816	1.7159
41	NC_007432	Streptococcus agalactiae A909	2127839	1.7154
42	NC_003028	Streptococcus pneumoniae TIGR4	2160837	1.6901
43	NC_002978	Wolbachia endosymbiont of Drosophila melanogaster	1267782	1.6783
44	NC_006448	Streptococcus thermophilus LMG 18311	1796846	1.6730
45	NC_005362	Lactobacillus johnsonii NCC 533	1992676	1.6655
46	NC_006513	Azoarcus sp. EbN1	4296230	1.6633
47	NC_002946	Neisseria gonorrhoeae FA 1090	2153922	1.6629
48	NC_002929	Bordetella pertussis Tohama I	4086189	1.6622
49	NC_007954	Shewanella denitrificans OS217	4545906	1.6284
50	NC_004741	Shigella flexneri 2a str. 2457T	4599354	1.6136
51	NC_007164	Corynebacterium jeikeium K411	2462499	1.6055
52	NC_003198	Salmonella enterica subsp. enterica serovar Typhi str. CT18	4809037	1.5869
53	NC_002662	Lactococcus lactis subsp. lactis Il1403	2365589	1.5586
54	NC_003454	Fusobacterium nucleatum subsp. nucleatum ATCC 25586	2174500	1.5457
55	NC_004572	Tropheryma whipplei str. Twist	927303	1.5277
56	NC_004350	Streptococcus mutans UA159	2030921	1.5233
57	NC_000915	Helicobacter pylori 26695	1667867	1.5229
58	NC_006834	Xanthomonas oryzae pv. oryzae KACC10331	4941439	1.5119
59	NC_002976	Staphylococcus epidermidis RP62A	2616530	1.5083
60	NC_004088	Yersinia pestis KIM	4600755	1.4926
61	NC_002163	Campylobacter jejuni subsp. jejuni NCTC 11168	1641481	1.4724
62	NC_005090	Wolinella succinogenes DSM 1740	2110355	1.4649
63	NC_007576	Lactobacillus sakei subsp. sakei 23K	1884661	1.4584
64	NC_004347	Shewanella oneidensis MR-1	4969803	1.4534
65	NC_003869	Thermoanaerobacter tengcongensis MB4	2689445	1.4486
66	NC_004369	Corynebacterium efficiens YS-314	3147090	1.4432
67	NC_003098	Streptococcus pneumoniae R6	2038615	1.4171
68	NC_004578	Pseudomonas syringae pv. tomato str. DC3000	6397126	1.4096
69	NC_007955	Methanococcoides burtonii DSM 6242	2575032	1.4029
70	NC_003912	Campylobacter jejuni RM1221	1777831	1.3956
71	NC_007520	Thiomicrospira crunogena XCL-2	2427734	1.3829
72	NC_007146	Haemophilus influenzae 86-028NP	1913428	1.3796
73	NC_007168	Staphylococcus haemolyticus JCSC1435	2685015	1.3731
74	NC_007204	Psychrobacter arcticus 273-4	2650701	1.3618
75	NC_007406	Nitrobacter winogradskyi Nb-255	3402093	1.3616
76	NC_006370	Photobacterium profundum SS9	4085304	1.3569
77	NC_007512	Pelodictyon luteolum DSM 273	2364842	1.3541
78	NC_004307	Bifidobacterium longum NCC2705	2256640	1.3404
79	NC_002754	Sulfolobus solfataricus P2	2992245	1.3393
80	NC_007716	Aster yellows witches'-broom phytoplasma AYWB	706569	1.3377
81	NC_006087	Leifsonia xyli subsp. xyli str. CTCB07	2584158	1.3358
82	NC_002758	Staphylococcus aureus subsp. aureus Mu50	2878529	1.3249
83	NC_003143	Yersinia pestis CO92	4653728	1.3248
84	NC_002952	Staphylococcus aureus subsp. aureus MRSA252	2902619	1.3114
85	NC_002570	Bacillus halodurans C-125	4202352	1.3077
86	NC_005810	Yersinia pestis biovar Medievalis str. 91001	4595065	1.3053
87	NC_004344	Wigglesworthia glossinidia endosymbiont of Glossina brevipalpis	697724	1.3027
88	NC_004631	Salmonella enterica subsp. enterica serovar Typhi Ty2	4791961	1.2962
89	NC_004310	Brucella suis 1330	2107794	1.2949
90	NC_006055	Mesoplasma florum L1	793224	1.2860
91	NC_003888	Streptomyces coelicolor A3(2)	8667507	1.2834
92	NC_007929	Lactobacillus salivarius subsp. salivarius UCC118	1827111	1.2714
93	NC_002935	Corynebacterium diphtheriae NCTC 13129	2488635	1.2569
94	NC_007429	Chlamydia trachomatis A/HAR-13	1044459	1.2527
95	NC_004193	Oceanobacillus iheyensis HTE831	3630528	1.2412
96	NC_007796	Methanospirillum hungatei JF-1	3544738	1.2400
Continued on next page				

Prokaryote I_r sorted by I_r, continued from previous page				
No.	Accession	Organism	Size	I_r
97	NC_005773	Pseudomonas syringae pv. phaseolicola 1448A	5928787	1.2336
98	NC_007614	Nitrosospira multiformis ATCC 25196	3184243	1.2264
99	NC_006932	Brucella abortus biovar 1 str. 9-941	2124241	1.2179
100	NC_007777	Frankia sp. CcI3	5433628	1.2133
101	NC_003552	Methanosarcina acetivorans C2A	5751492	1.2088
102	NC_005966	Acinetobacter sp. ADP1	3598621	1.1968
103	NC_002973	Listeria monocytogenes str. 4b F2365	2905187	1.1968
104	NC_006510	Geobacillus kaustophilus HTA426	3544776	1.1875
105	NC_006526	Zymomonas mobilis subsp. mobilis ZM4	2056416	1.1722
106	NC_002737	Streptococcus pyogenes M1 GAS	1852441	1.1711
107	NC_003910	Colwellia psychrerythraea 34H	5373180	1.1612
108	NC_003047	Sinorhizobium meliloti 1021	3654135	1.1582
109	NC_002977	Methylococcus capsulatus str. Bath	3304561	1.1542
110	NC_004547	Erwinia carotovora subsp. atroseptica SCRI1043	5064019	1.1497
111	NC_005085	Chromobacterium violaceum ATCC 12472	4751080	1.1450
112	NC_004603	Vibrio parahaemolyticus RIMD 2210633	3288558	1.1373
113	NC_007964	Nitrobacter hamburgensis X14	4406967	1.1275
114	NC_006677	Gluconobacter oxydans 621H	2702173	1.1275
115	NC_007481	Pseudoalteromonas haloplanktis TAC125	3214944	1.1189
116	NC_006177	Symbiobacterium thermophilum IAM 14863	3566135	1.1146
117	NC_000117	Chlamydia trachomatis D/UW-3/CX	1042519	1.1124
118	NC_006511	Salmonella enterica subsp. enterica serovar Paratyphi A str. ATCC 9150	4585229	1.1117
119	NC_007413	Anabaena variabilis ATCC 29413	6365727	1.0871
120	NC_003210	Listeria monocytogenes EGD-e	2944528	1.0798
121	NC_007626	Magnetospirillum magneticum AMB-1	4967148	1.0752
122	NC_003197	Salmonella typhimurium LT2	4857432	1.0644
123	NC_006300	Mannheimia succiniciproducens MBEL55E	2314078	1.0513
124	NC_002928	Bordetella parapertussis 12822	4773551	1.0371
125	NC_004431	Escherichia coli CFT073	5231428	1.0265
126	NC_007517	Geobacter metallireducens GS-15	3997420	1.0213
127	NC_004459	Vibrio vulnificus CMCP6	3281945	1.0189
128	NC_007332	Mycoplasma hyopneumoniae 7448	920079	1.0156
129	NC_007298	Dechloromonas aromatica RCB	4501104	1.0127
130	NC_003911	Silicibacter pomeroyi DSS-3	4109442	1.0099
131	NC_007651	Burkholderia thailandensis E264	3809201	1.0086
132	NC_005071	Prochlorococcus marinus str. MIT 9313	2410873	1.0086
133	NC_006840	Vibrio fischeri ES114	2906179	0.9957
134	NC_007109	Rickettsia felis URRWXCal2	1485148	0.9924
135	NC_002663	Pasteurella multocida subsp. multocida str. Pm70	2257487	0.9920
136	NC_006905	Salmonella enterica subsp. enterica serovar Choleraesuis str. SC-B67	4755700	0.9917
137	NC_007761	Rhizobium etli CFN 42	4381608	0.9900
138	NC_004113	Thermosynechococcus elongatus BP-1	2593857	0.9895
139	NC_000918	Aquifex aeolicus VF5	1551335	0.9886
140	NC_006274	Bacillus cereus E33L	5300915	0.9827
141	NC_004722	Bacillus cereus ATCC 14579	5411809	0.9823
142	NC_005139	Vibrio vulnificus YJ016	3354505	0.9815
143	NC_004557	Clostridium tetani E88	2799251	0.9781
144	NC_002505	Vibrio cholerae O1 biovar eltor str. N16961	2961149	0.9737
145	NC_003450	Corynebacterium glutamicum ATCC 13032	3309401	0.9734
146	NC_005791	Methanococcus maripaludis S2	1661137	0.9724
147	NC_006833	Wolbachia endosymbiont strain TRS of Brugia malayi	1080084	0.9679
148	NC_003295	Ralstonia solanacearum GMI1000	3716413	0.9614
149	NC_003901	Methanosarcina mazei Go1	4096345	0.9588
150	NC_006369	Legionella pneumophila str. Lens	3345687	0.9554
151	NC_000921	Helicobacter pylori J99	1643831	0.9450
152	NC_003902	Xanthomonas campestris pv. campestris str. ATCC 33913	5076188	0.9445
153	NC_003030	Clostridium acetobutylicum ATCC 824	3940880	0.9370
Continued on next page				

Prokaryote I_r sorted by I_r, continued from previous page				
No.	Accession	Organism	Size	I_r
154	NC_007513	Synechococcus sp. CC9902	2234828	0.9351
155	NC_007519	Desulfovibrio desulfuricans G20	3730232	0.9349
156	NC_000919	Treponema pallidum subsp. pallidum str. Nichols	1138011	0.9307
157	NC_007516	Synechococcus sp. CC9605	2510659	0.9301
158	NC_005957	Bacillus thuringiensis serovar konkukian str. 97-27	5237682	0.9296
159	NC_002947	Pseudomonas putida KT2440	6181863	0.9178
160	NC_007775	Synechococcus sp. JA-3-3Ab	2932766	0.8916
161	NC_004663	Bacteroides thetaiotaomicron VPI-5482	6260361	0.8864
162	NC_004668	Enterococcus faecalis V583	3218031	0.8853
163	NC_007681	Methanosphaera stadtmanae DSM 3091	1767403	0.8824
164	NC_007350	Staphylococcus saprophyticus subsp. saprophyticus ATCC 15305	2516575	0.8694
165	NC_002939	Geobacter sulfurreducens PCA	3814139	0.8665
166	NC_003304	Agrobacterium tumefaciens str. C58	2841490	0.8590
167	NC_007294	Mycoplasma synoviae 53	799476	0.8568
168	NC_006958	Corynebacterium glutamicum ATCC 13032	3282708	0.8566
169	NC_006155	Yersinia pseudotuberculosis IP 32953	4744671	0.8542
170	NC_007645	Hahella chejuensis KCTC 2396	7215267	0.8519
171	NC_004842	Anaplasma marginale str. St. Maries	1197687	0.8497
172	NC_007907	Desulfitobacterium hafniense Y51	5727534	0.8341
173	NC_002677	Mycobacterium leprae TN	3268203	0.8330
174	NC_002937	Desulfovibrio vulgaris subsp. vulgaris str. Hildenborough	3570858	0.8278
175	NC_007354	Ehrlichia canis str. Jake	1315030	0.8271
176	NC_007952	Burkholderia xenovorans LB400	3363523	0.8259
177	NC_002620	Chlamydia muridarum Nigg	1072950	0.8180
178	NC_006322	Bacillus licheniformis ATCC 14580	4222645	0.8166
179	NC_006368	Legionella pneumophila str. Paris	3503610	0.8133
180	NC_006512	Idiomarina loihiensis L2TR	2839318	0.8116
181	NC_002516	Pseudomonas aeruginosa PAO1	6264403	0.8098
182	NC_007794	Novosphingobium aromaticivorans DSM 12444	3561584	0.8086
183	NC_003228	Bacteroides fragilis NCTC 9343	5205140	0.8064
184	NC_007940	Rickettsia bellii RML369-C	1522076	0.7991
185	NC_007492	Pseudomonas fluorescens PfO-1	6438405	0.7972
186	NC_004829	Mycoplasma gallisepticum R	996422	0.7969
187	NC_007086	Xanthomonas campestris pv. campestris str. 8004	5148708	0.7958
188	NC_004432	Mycoplasma penetrans HF-2	1358633	0.7897
189	NC_002971	Coxiella burnetii RSA 493	1995281	0.7864
190	NC_000911	Synechocystis sp. PCC 6803	3573470	0.7764
191	NC_007498	Pelobacter carbinolicus DSM 2380	3665893	0.7718
192	AC_000091	Escherichia coli W3110	4646332	0.7717
193	NC_002951	Staphylococcus aureus subsp. aureus COL	2809422	0.7696
194	NC_003317	Brucella melitensis 16M	2117144	0.7690
195	NC_003997	Bacillus anthracis str. Ames	5227293	0.7686
196	NC_003909	Bacillus cereus ATCC 10987	5224283	0.7650
197	NC_004461	Staphylococcus epidermidis ATCC 12228	2499279	0.7623
198	NC_004567	Lactobacillus plantarum WCFS1	3308274	0.7578
199	NC_007946	Escherichia coli UTI89	5065741	0.7572
200	NC_006138	Desulfotalea psychrophila LSv54	3523383	0.7555
201	NC_005945	Bacillus anthracis str. Sterne	5228663	0.7502
202	NC_007622	Staphylococcus aureus RF122	2742531	0.7407
203	NC_007530	Bacillus anthracis str. 'Ames Ancestor'	5227419	0.7353
204	NC_007908	Rhodoferax ferrireducens DSM 15236	4712337	0.7239
205	NC_006350	Burkholderia pseudomallei K96243	4074542	0.7214
206	NC_007618	Brucella melitensis biovar Abortus 2308	2121359	0.7144
207	NC_000913	Escherichia coli K12	4639675	0.7124
208	NC_003272	Nostoc sp. PCC 7120	6413771	0.7108
209	NC_002942	Legionella pneumophila subsp. pneumophila str. Philadelphia 1	3397754	0.7081
210	NC_007759	Syntrophus aciditrophicus SB	3179300	0.7056
Continued on next page				

Prokaryote I_r sorted by I_r, continued from previous page				
No.	Accession	Organism	Size	I_r
211	NC_002771	Mycoplasma pulmonis UAB CTIP	963879	0.7040
212	NC_007604	Synechococcus elongatus PCC 7942	2695903	0.6997
213	NC_007643	Rhodospirillum rubrum ATCC 11170	4352825	0.6971
214	NC_002696	Caulobacter crescentus CB15	4016947	0.6965
215	NC_006814	Lactobacillus acidophilus NCFM	1993564	0.6904
216	NC_003919	Xanthomonas axonopodis pv. citri str. 306	5175554	0.6897
217	NC_003923	Staphylococcus aureus subsp. aureus MW2	2820462	0.6857
218	NC_006576	Synechococcus elongatus PCC 6301	2696255	0.6855
219	NC_007503	Carboxydothermus hydrogenoformans Z-2901	2401520	0.6723
220	NC_006347	Bacteroides fragilis YCH46	5277274	0.6711
221	NC_003366	Clostridium perfringens str. 13	3031430	0.6648
222	NC_006461	Thermus thermophilus HB8	1849742	0.6630
223	NC_002953	Staphylococcus aureus subsp. aureus MSSA476	2799802	0.6539
224	NC_002932	Chlorobium tepidum TLS	2154946	0.6508
225	NC_007508	Xanthomonas campestris pv. vesicatoria str. 85-10	5178466	0.6424
226	NC_007793	Staphylococcus aureus subsp. aureus USA300	2872769	0.6384
227	NC_007355	Methanosarcina barkeri str. fusaro	4837408	0.6314
228	NC_007404	Thiobacillus denitrificans ATCC 25259	2909809	0.6284
229	NC_002944	Mycobacterium avium subsp. paratuberculosis K-10	4829781	0.6248
230	NC_007333	Thermobifida fusca YX	3642249	0.6165
231	NC_007295	Mycoplasma hyopneumoniae J	897405	0.6136
232	NC_004342	Leptospira interrogans serovar Lai str. 56601	4332241	0.6134
233	NC_005070	Synechococcus sp. WH 8102	2434428	0.6132
234	NC_006270	Bacillus licheniformis ATCC 14580	4222334	0.6127
235	NC_003155	Streptomyces avermitilis MA-4680	9025608	0.6095
236	NC_007776	Synechococcus sp. JA-2-3B'a(2-13)	3046682	0.6063
237	NC_004463	Bradyrhizobium japonicum USDA 110	9105828	0.6055
238	NC_003062	Agrobacterium tumefaciens str. C58	2841581	0.5977
239	NC_004129	Pseudomonas fluorescens Pf-5	7074893	0.5945
240	NC_007005	Pseudomonas syringae pv. syringae B728a	6093698	0.5924
241	NC_001263	Deinococcus radiodurans R1	2648638	0.5870
242	NC_002678	Mesorhizobium loti MAFF303099	7036071	0.5815
243	NC_007514	Chlorobium chlorochromatii CaD3	2572079	0.5804
244	NC_007795	Staphylococcus aureus subsp. aureus NCTC 8325	2821361	0.5725
245	NC_007434	Burkholderia pseudomallei 1710b	4126292	0.5723
246	NC_007925	Rhodopseudomonas palustris BisB18	5513844	0.5676
247	NC_000964	Bacillus subtilis subsp. subtilis str. 168	4214630	0.5659
248	NC_000962	Mycobacterium tuberculosis H37Rv	4411532	0.5647
249	NC_006156	Borrelia garinii PBi	904246	0.5534
250	NC_006085	Propionibacterium acnes KPA171202	2560265	0.5514
251	NC_007484	Nitrosococcus oceani ATCC 19707	3481691	0.5504
252	NC_002967	Treponema denticola ATCC 35405	2843201	0.5407
253	NC_007958	Rhodopseudomonas palustris BisB5	4892717	0.5391
254	NC_007963	Chromohalobacter salexigens DSM 3043	3696649	0.5310
255	NC_005027	Rhodopirellula baltica SH 1	7145576	0.5292
256	NC_005835	Thermus thermophilus HB27	1894877	0.5242
257	NC_002162	Ureaplasma parvum serovar 3 str. ATCC 700970	751719	0.5090
258	NC_007644	Moorella thermoacetica ATCC 39073	2628784	0.5089
259	NC_005823	Leptospira interrogans serovar Copenhageni str. Fiocruz L1-130	4277185	0.4965
260	NC_007912	Saccharophagus degradans 2-40	5057531	0.4931
261	NC_005861	Candidatus Protochlamydia amoebophila UWE25	2414465	0.4693
262	NC_005363	Bdellovibrio bacteriovorus HD100	3782950	0.4658
263	NC_002755	Mycobacterium tuberculosis CDC1551	4403837	0.4530
264	NC_007348	Ralstonia eutropha JMP134	2726152	0.4505
265	NC_006582	Bacillus clausii KSM-K16	4303871	0.4499
266	NC_005296	Rhodopseudomonas palustris CGA009	5459213	0.4375
267	NC_000916	Methanothermobacter thermautotrophicus str. Delta H	1751377	0.4256
Continued on next page				

Prokaryote I_r sorted by I_r, continued from previous page				
No.	Accession	Organism	Size	I_r
268	NC_006908	Mycoplasma mobile 163K	777079	0.4222
269	NC_007799	Ehrlichia chaffeensis str. Arkansas	1176248	0.4214
270	NC_006624	Thermococcus kodakarensis KOD1	2088737	0.4197
271	NC_002945	Mycobacterium bovis AF2122/97	4345492	0.4082
272	NC_003413	Pyrococcus furiosus DSM 3638	1908256	0.3974
273	NC_004917	Helicobacter hepaticus ATCC 51449	1799146	0.3778
274	NC_007633	Mycoplasma capricolum subsp. capricolum ATCC 27343	1010023	0.3692
275	NC_005125	Gloeobacter violaceus PCC 7421	4659019	0.3608
276	NC_003106	Sulfolobus tokodaii str. 7	2694756	0.3584
277	NC_003364	Pyrobaculum aerophilum str. IM2	2222430	0.3478
278	NC_004552	Chlamydophila abortus S26/3	1144377	0.3441
279	NC_006360	Mycoplasma hyopneumoniae 232	892758	0.3422
280	NC_001318	Borrelia burgdorferi B31	910724	0.3405
281	NC_000912	Mycoplasma pneumoniae M129	816394	0.3394
282	NC_007335	Prochlorococcus marinus str. NATL2A	1842899	0.3312
283	NC_002607	Halobacterium sp. NRC-1	2014239	0.3272
284	NC_005295	Ehrlichia ruminantium str. Welgevonden	1516355	0.3254
285	NC_000917	Archaeoglobus fulgidus DSM 4304	2178400	0.3239
286	NC_007760	Anaeromyxobacter dehalogenans 2CP-C	5013479	0.3135
287	NC_007948	Polaromonas sp. JS666	5200264	0.3134
288	NC_002179	Chlamydophila pneumoniae AR39	1229853	0.3040
289	NC_000909	Methanocaldococcus jannaschii DSM 2661	1664970	0.2940
290	NC_005043	Chlamydophila pneumoniae TW-183	1225935	0.2793
291	NC_006831	Ehrlichia ruminantium str. Gardel	1499920	0.2770
292	NC_003361	Chlamydophila caviae GPIC	1173390	0.2669
293	NC_006361	Nocardia farcinica IFM 10152	6021225	0.2595
294	NC_006832	Ehrlichia ruminantium str. Welgevonden	1512977	0.2471
295	NC_006396	Haloarcula marismortui ATCC 43049	3131724	0.2318
296	NC_000922	Chlamydophila pneumoniae CWL029	1230230	0.2283
297	NC_007722	Erythrobacter litoralis HTCC2594	3052398	0.2031
298	NC_007356	Dehalococcoides sp. CBDB1	1395502	0.1714
299	NC_007677	Salinibacter ruber DSM 13855	3551823	0.1685
300	NC_002689	Thermoplasma volcanium GSS1	1584804	0.1651
301	NC_007802	Jannaschia sp. CCS1	4317977	0.1633
302	NC_000853	Thermotoga maritima MSB8	1860725	0.1608
303	NC_007577	Prochlorococcus marinus str. MIT 9312	1709204	0.1503
304	NC_000908	Mycoplasma genitalium G37	580074	0.1377
305	NC_007426	Natronomonas pharaonis DSM 2160	2595221	0.1352
306	NC_002491	Chlamydophila pneumoniae J138	1226565	0.1349
307	NC_007778	Rhodopseudomonas palustris HaA2	5331656	0.1193
308	NC_005072	Prochlorococcus marinus subsp. pastoris str. CCMP1986	1657990	0.1149
309	NC_000854	Aeropyrum pernix K1	1669695	0.1048
310	NC_003103	Rickettsia conorii str. Malish 7	1268755	0.0968
311	NC_007493	Rhodobacter sphaeroides 2.4.1	3188609	0.0892
312	NC_007509	Burkholderia sp. 383	1395069	0.0882
313	NC_000961	Pyrococcus horikoshii OT3	1738505	0.0856
314	NC_007205	Candidatus Pelagibacter ubique HTCC1062	1308759	0.0787
315	NC_000868	Pyrococcus abyssi GE5	1765118	0.0713
316	NC_005877	Picrophilus torridus DSM 9790	1545895	0.0702
317	NC_006142	Rickettsia typhi str. Wilmington	1111496	0.0607
318	NC_002578	Thermoplasma acidophilum DSM 1728	1564906	0.0577
319	NC_003551	Methanopyrus kandleri AV19	1694969	0.0532
320	NC_000963	Rickettsia prowazekii str. Madrid E	1111523	0.0514
321	NC_005213	Nanoarchaeum equitans Kin4-M	490885	0.0482
322	NC_007798	Neorickettsia sennetsu str. Miyayama	859006	0.0452
323	NC_007181	Sulfolobus acidocaldarius DSM 639	2225959	0.0428
324	NC_005042	Prochlorococcus marinus subsp. marinus str. CCMP1375	1751080	0.0373
Continued on next page				

Prokaryote I_r sorted by I_r, continued from previous page				
No.	Accession	Organism	Size	I_r
325	NC_005061	Candidatus Blochmannia floridanus	705557	0.0325
326	NC_007292	Candidatus Blochmannia pennsylvanicus str. BPEN	791654	0.0293
327	NC_004061	Buchnera aphidicola str. Sg (Schizaphis graminum)	641454	0.0293
328	NC_007899	Chlamydophila felis Fe/C-56	1166239	0.0284
329	NC_002528	Buchnera aphidicola str. APS (Acyrthosiphon pisum)	640681	0.0253
330	NC_004545	Buchnera aphidicola str. Bp (Baizongia pistaciae)	615980	0.0189

Table 2: I_r values for prokaryotes sorted alphabetically

No.	Accession	Organism	Size	I_r
1	NC_005966	Acinetobacter sp. ADP1	3598621	1.1968
2	NC_000854	Aeropyrum pernix K1	1669695	0.1048
3	NC_003304	Agrobacterium tumefaciens str. C58	2841490	0.8590
4	NC_003062	Agrobacterium tumefaciens str. C58	2841581	0.5977
5	NC_007413	Anabaena variabilis ATCC 29413	6365727	1.0871
6	NC_007760	Anaeromyxobacter dehalogenans 2CP-C	5013479	0.3135
7	NC_004842	Anaplasma marginale str. St. Maries	1197687	0.8497
8	NC_007797	Anaplasma phagocytophilum HZ	1471282	1.7544
9	NC_000918	Aquifex aeolicus VF5	1551335	0.9886
10	NC_000917	Archaeoglobus fulgidus DSM 4304	2178400	0.3239
11	NC_007716	Aster yellows witches'-broom phytoplasma AYWB	706569	1.3377
12	NC_006513	Azoarcus sp. EbN1	4296230	1.6633
13	NC_007530	Bacillus anthracis str. 'Ames Ancestor'	5227419	0.7353
14	NC_003997	Bacillus anthracis str. Ames	5227293	0.7686
15	NC_005945	Bacillus anthracis str. Sterne	5228663	0.7502
16	NC_003909	Bacillus cereus ATCC 10987	5224283	0.7650
17	NC_004722	Bacillus cereus ATCC 14579	5411809	0.9823
18	NC_006274	Bacillus cereus E33L	5300915	0.9827
19	NC_006582	Bacillus clausii KSM-K16	4303871	0.4499
20	NC_002570	Bacillus halodurans C-125	4202352	1.3077
21	NC_006270	Bacillus licheniformis ATCC 14580	4222334	0.6127
22	NC_006322	Bacillus licheniformis ATCC 14580	4222645	0.8166
23	NC_000964	Bacillus subtilis subsp. subtilis str. 168	4214630	0.5659
24	NC_005957	Bacillus thuringiensis serovar konkukian str. 97-27	5237682	0.9296
25	NC_003228	Bacteroides fragilis NCTC 9343	5205140	0.8064
26	NC_006347	Bacteroides fragilis YCH46	5277274	0.6711
27	NC_004663	Bacteroides thetaiotaomicron VPI-5482	6260361	0.8864
28	NC_005956	Bartonella henselae str. Houston-1	1931047	2.3482
29	NC_005955	Bartonella quintana str. Toulouse	1581384	1.9798
30	NC_005363	Bdellovibrio bacteriovorus HD100	3782950	0.4658
31	NC_004307	Bifidobacterium longum NCC2705	2256640	1.3404
32	NC_002927	Bordetella bronchiseptica RB50	5339179	1.7593
33	NC_002928	Bordetella parapertussis 12822	4773551	1.0371
34	NC_002929	Bordetella pertussis Tohama I	4086189	1.6622
35	NC_001318	Borrelia burgdorferi B31	910724	0.3405
36	NC_006156	Borrelia garinii PBi	904246	0.5534
37	NC_004463	Bradyrhizobium japonicum USDA 110	9105828	0.6055
38	NC_006932	Brucella abortus biovar 1 str. 9-941	2124241	1.2179
39	NC_003317	Brucella melitensis 16M	2117144	0.7690
40	NC_007618	Brucella melitensis biovar Abortus 2308	2121359	0.7144
41	NC_004310	Brucella suis 1330	2107794	1.2949
42	NC_002528	Buchnera aphidicola str. APS (Acyrthosiphon pisum)	640681	0.0253
43	NC_004545	Buchnera aphidicola str. Bp (Baizongia pistaciae)	615980	0.0189
44	NC_004061	Buchnera aphidicola str. Sg (Schizaphis graminum)	641454	0.0293
45	NC_006349	Burkholderia mallei ATCC 23344	2325379	1.9111
46	NC_007434	Burkholderia pseudomallei 1710b	4126292	0.5723
47	NC_006350	Burkholderia pseudomallei K96243	4074542	0.7214
48	NC_007509	Burkholderia sp. 383	1395069	0.0882
49	NC_007651	Burkholderia thailandensis E264	3809201	1.0086
50	NC_007952	Burkholderia xenovorans LB400	3363523	0.8259
51	NC_003912	Campylobacter jejuni RM1221	1777831	1.3956
52	NC_002163	Campylobacter jejuni subsp. jejuni NCTC 11168	1641481	1.4724
53	NC_005061	Candidatus Blochmannia floridanus	705557	0.0325
54	NC_007292	Candidatus Blochmannia pennsylvanicus str. BPEN	791654	0.0293
55	NC_007205	Candidatus Pelagibacter ubique HTCC1062	1308759	0.0787

Continued on next page

Prokaryote I_r sorted alphabetically, continued from previous page				
No.	Accession	Organism	Size	I_r
56	NC_005861	Candidatus Protochlamydia amoebophila UWE25	2414465	0.4693
57	NC_007503	Carboxydothermus hydrogenoformans Z-2901	2401520	0.6723
58	NC_002696	Caulobacter crescentus CB15	4016947	0.6965
59	NC_002620	Chlamydia muridarum Nigg	1072950	0.8180
60	NC_007429	Chlamydia trachomatis A/HAR-13	1044459	1.2527
61	NC_000117	Chlamydia trachomatis D/UW-3/CX	1042519	1.1124
62	NC_004552	Chlamydophila abortus S26/3	1144377	0.3441
63	NC_003361	Chlamydophila caviae GPIC	1173390	0.2669
64	NC_007899	Chlamydophila felis Fe/C-56	1166239	0.0284
65	NC_002179	Chlamydophila pneumoniae AR39	1229853	0.3040
66	NC_000922	Chlamydophila pneumoniae CWL029	1230230	0.2283
67	NC_002491	Chlamydophila pneumoniae J138	1226565	0.1349
68	NC_005043	Chlamydophila pneumoniae TW-183	1225935	0.2793
69	NC_007514	Chlorobium chlorochromatii CaD3	2572079	0.5804
70	NC_002932	Chlorobium tepidum TLS	2154946	0.6508
71	NC_005085	Chromobacterium violaceum ATCC 12472	4751080	1.1450
72	NC_007963	Chromohalobacter salexigens DSM 3043	3696649	0.5310
73	NC_003030	Clostridium acetobutylicum ATCC 824	3940880	0.9370
74	NC_003366	Clostridium perfringens str. 13	3031430	0.6648
75	NC_004557	Clostridium tetani E88	2799251	0.9781
76	NC_003910	Colwellia psychrerythraea 34H	5373180	1.1612
77	NC_002935	Corynebacterium diphtheriae NCTC 13129	2488635	1.2569
78	NC_004369	Corynebacterium efficiens YS-314	3147090	1.4432
79	NC_006958	Corynebacterium glutamicum ATCC 13032	3282708	0.8566
80	NC_003450	Corynebacterium glutamicum ATCC 13032	3309401	0.9734
81	NC_007164	Corynebacterium jeikeium K411	2462499	1.6055
82	NC_002971	Coxiella burnetii RSA 493	1995281	0.7864
83	NC_007298	Dechloromonas aromatica RCB	4501104	1.0127
84	NC_002936	Dehalococcoides ethenogenes 195	1469720	4.0257
85	NC_007356	Dehalococcoides sp. CBDB1	1395502	0.1714
86	NC_001263	Deinococcus radiodurans R1	2648638	0.5870
87	NC_007907	Desulfitobacterium hafniense Y51	5727534	0.8341
88	NC_006138	Desulfotalea psychrophila LSv54	3523383	0.7555
89	NC_007519	Desulfovibrio desulfuricans G20	3730232	0.9349
90	NC_002937	Desulfovibrio vulgaris subsp. vulgaris str. Hildenborough	3570858	0.8278
91	NC_007354	Ehrlichia canis str. Jake	1315030	0.8271
92	NC_007799	Ehrlichia chaffeensis str. Arkansas	1176248	0.4214
93	NC_006831	Ehrlichia ruminantium str. Gardel	1499920	0.2770
94	NC_006832	Ehrlichia ruminantium str. Welgevonden	1512977	0.2471
95	NC_005295	Ehrlichia ruminantium str. Welgevonden	1516355	0.3254
96	NC_004668	Enterococcus faecalis V583	3218031	0.8853
97	NC_004547	Erwinia carotovora subsp. atroseptica SCRI1043	5064019	1.1497
98	NC_007722	Erythrobacter litoralis HTCC2594	3052398	0.2031
99	NC_004431	Escherichia coli CFT073	5231428	1.0265
100	NC_000913	Escherichia coli K12	4639675	0.7124
101	NC_002655	Escherichia coli O157:H7 EDL933	5528445	3.5209
102	NC_002695	Escherichia coli O157:H7	5498450	1.9753
103	NC_007946	Escherichia coli UTI89	5065741	0.7572
104	AC_000091	Escherichia coli W3110	4646332	0.7717
105	NC_007880	Francisella tularensis subsp. holarctica	1895994	3.7227
106	NC_006570	Francisella tularensis subsp. tularensis SCHU S4	1892819	3.9503
107	NC_007777	Frankia sp. CcI3	5433628	1.2133
108	NC_003454	Fusobacterium nucleatum subsp. nucleatum ATCC 25586	2174500	1.5457
109	NC_006510	Geobacillus kaustophilus HTA426	3544776	1.1875
110	NC_007517	Geobacter metallireducens GS-15	3997420	1.0213
111	NC_002939	Geobacter sulfurreducens PCA	3814139	0.8665
112	NC_005125	Gloeobacter violaceus PCC 7421	4659019	0.3608
Continued on next page				

Prokaryote I_r sorted alphabetically, continued from previous page				
No.	Accession	Organism	Size	I_r
113	NC_006677	Gluconobacter oxydans 621H	2702173	1.1275
114	NC_002940	Haemophilus ducreyi 35000HP	1698955	2.8218
115	NC_007146	Haemophilus influenzae 86-028NP	1913428	1.3796
116	NC_000907	Haemophilus influenzae Rd KW20	1830138	1.8248
117	NC_007645	Hahella chejuensis KCTC 2396	7215267	0.8519
118	NC_006396	Haloarcula marismortui ATCC 43049	3131724	0.2318
119	NC_002607	Halobacterium sp. NRC-1	2014239	0.3272
120	NC_004917	Helicobacter hepaticus ATCC 51449	1799146	0.3778
121	NC_000915	Helicobacter pylori 26695	1667867	1.5229
122	NC_000921	Helicobacter pylori J99	1643831	0.9450
123	NC_006512	Idiomarina loihiensis L2TR	2839318	0.8116
124	NC_007802	Jannaschia sp. CCS1	4317977	0.1633
125	NC_006814	Lactobacillus acidophilus NCFM	1993564	0.6904
126	NC_005362	Lactobacillus johnsonii NCC 533	1992676	1.6655
127	NC_004567	Lactobacillus plantarum WCFS1	3308274	0.7578
128	NC_007576	Lactobacillus sakei subsp. sakei 23K	1884661	1.4584
129	NC_007929	Lactobacillus salivarius subsp. salivarius UCC118	1827111	1.2714
130	NC_002662	Lactococcus lactis subsp. lactis Il1403	2365589	1.5586
131	NC_006369	Legionella pneumophila str. Lens	3345687	0.9554
132	NC_006368	Legionella pneumophila str. Paris	3503610	0.8133
133	NC_002942	Legionella pneumophila subsp. pneumophila str. Philadelphia 1	3397754	0.7081
134	NC_006087	Leifsonia xyli subsp. xyli str. CTCB07	2584158	1.3358
135	NC_005823	Leptospira interrogans serovar Copenhageni str. Fiocruz L1-130	4277185	0.4965
136	NC_004342	Leptospira interrogans serovar Lai str. 56601	4332241	0.6134
137	NC_003212	Listeria innocua Clip11262	3011208	1.8736
138	NC_003210	Listeria monocytogenes EGD-e	2944528	1.0798
139	NC_002973	Listeria monocytogenes str. 4b F2365	2905187	1.1968
140	NC_007626	Magnetospirillum magneticum AMB-1	4967148	1.0752
141	NC_006300	Mannheimia succiniciproducens MBEL55E	2314078	1.0513
142	NC_006055	Mesoplasma florum L1	793224	1.2860
143	NC_002678	Mesorhizobium loti MAFF303099	7036071	0.5815
144	NC_000909	Methanocaldococcus jannaschii DSM 2661	1664970	0.2940
145	NC_007955	Methanococcoides burtonii DSM 6242	2575032	1.4029
146	NC_005791	Methanococcus maripaludis S2	1661137	0.9724
147	NC_003551	Methanopyrus kandleri AV19	1694969	0.0532
148	NC_003552	Methanosarcina acetivorans C2A	5751492	1.2088
149	NC_007355	Methanosarcina barkeri str. fusaro	4837408	0.6314
150	NC_003901	Methanosarcina mazei Go1	4096345	0.9588
151	NC_007681	Methanosphaera stadtmanae DSM 3091	1767403	0.8824
152	NC_007796	Methanospirillum hungatei JF-1	3544738	1.2400
153	NC_000916	Methanothermobacter thermautotrophicus str. Delta H	1751377	0.4256
154	NC_007947	Methylobacillus flagellatus KT	2971517	6.3367
155	NC_002977	Methylococcus capsulatus str. Bath	3304561	1.1542
156	NC_007644	Moorella thermoacetica ATCC 39073	2628784	0.5089
157	NC_002944	Mycobacterium avium subsp. paratuberculosis K-10	4829781	0.6248
158	NC_002945	Mycobacterium bovis AF2122/97	4345492	0.4082
159	NC_002677	Mycobacterium leprae TN	3268203	0.8330
160	NC_002755	Mycobacterium tuberculosis CDC1551	4403837	0.4530
161	NC_000962	Mycobacterium tuberculosis H37Rv	4411532	0.5647
162	NC_007633	Mycoplasma capricolum subsp. capricolum ATCC 27343	1010023	0.3692
163	NC_004829	Mycoplasma gallisepticum R	996422	0.7969
164	NC_000908	Mycoplasma genitalium G37	580074	0.1377
165	NC_006360	Mycoplasma hyopneumoniae 232	892758	0.3422
166	NC_007332	Mycoplasma hyopneumoniae 7448	920079	1.0156
167	NC_007295	Mycoplasma hyopneumoniae J	897405	0.6136
168	NC_006908	Mycoplasma mobile 163K	777079	0.4222
169	NC_005364	Mycoplasma mycoides subsp. mycoides SC str. PG1	1211703	2.9206

Continued on next page

Prokaryote I_r sorted alphabetically, continued from previous page				
No.	Accession	Organism	Size	I_r
170	NC_004432	Mycoplasma penetrans HF-2	1358633	0.7897
171	NC_000912	Mycoplasma pneumoniae M129	816394	0.3394
172	NC_002771	Mycoplasma pulmonis UAB CTIP	963879	0.7040
173	NC_007294	Mycoplasma synoviae 53	799476	0.8568
174	NC_005213	Nanoarchaeum equitans Kin4-M	490885	0.0482
175	NC_007426	Natronomonas pharaonis DSM 2160	2595221	0.1352
176	NC_002946	Neisseria gonorrhoeae FA 1090	2153922	1.6629
177	NC_003112	Neisseria meningitidis MC58	2272360	3.8422
178	NC_003116	Neisseria meningitidis Z2491	2184406	1.7507
179	NC_007798	Neorickettsia sennetsu str. Miyayama	859006	0.0452
180	NC_007964	Nitrobacter hamburgensis X14	4406967	1.1275
181	NC_007406	Nitrobacter winogradskyi Nb-255	3402093	1.3616
182	NC_007484	Nitrosococcus oceani ATCC 19707	3481691	0.5504
183	NC_004757	Nitrosomonas europaea ATCC 19718	2812094	1.7172
184	NC_007614	Nitrosospira multiformis ATCC 25196	3184243	1.2264
185	NC_006361	Nocardia farcinica IFM 10152	6021225	0.2595
186	NC_003272	Nostoc sp. PCC 7120	6413771	0.7108
187	NC_007794	Novosphingobium aromaticivorans DSM 12444	3561584	0.8086
188	NC_004193	Oceanobacillus iheyensis HTE831	3630528	1.2412
189	NC_005303	Onion yellows phytoplasma OY-M	860631	2.5371
190	NC_002663	Pasteurella multocida subsp. multocida str. Pm70	2257487	0.9920
191	NC_007498	Pelobacter carbinolicus DSM 2380	3665893	0.7718
192	NC_007512	Pelodictyon luteolum DSM 273	2364842	1.3541
193	NC_006370	Photobacterium profundum SS9	4085304	1.3569
194	NC_005126	Photorhabdus luminescens subsp. laumondii TTO1	5688987	1.8043
195	NC_005877	Picrophilus torridus DSM 9790	1545895	0.0702
196	NC_007948	Polaromonas sp. JS666	5200264	0.3134
197	NC_002950	Porphyromonas gingivalis W83	2343476	1.7821
198	NC_007577	Prochlorococcus marinus str. MIT 9312	1709204	0.1503
199	NC_005071	Prochlorococcus marinus str. MIT 9313	2410873	1.0086
200	NC_007335	Prochlorococcus marinus str. NATL2A	1842899	0.3312
201	NC_005042	Prochlorococcus marinus subsp. marinus str. CCMP1375	1751080	0.0373
202	NC_005072	Prochlorococcus marinus subsp. pastoris str. CCMP1986	1657990	0.1149
203	NC_006085	Propionibacterium acnes KPA171202	2560265	0.5514
204	NC_007481	Pseudoalteromonas haloplanktis TAC125	3214944	1.1189
205	NC_002516	Pseudomonas aeruginosa PAO1	6264403	0.8098
206	NC_004129	Pseudomonas fluorescens Pf-5	7074893	0.5945
207	NC_007492	Pseudomonas fluorescens PfO-1	6438405	0.7972
208	NC_002947	Pseudomonas putida KT2440	6181863	0.9178
209	NC_005773	Pseudomonas syringae pv. phaseolicola 1448A	5928787	1.2336
210	NC_007005	Pseudomonas syringae pv. syringae B728a	6093698	0.5924
211	NC_004578	Pseudomonas syringae pv. tomato str. DC3000	6397126	1.4096
212	NC_007204	Psychrobacter arcticus 273-4	2650701	1.3618
213	NC_003364	Pyrobaculum aerophilum str. IM2	2222430	0.3478
214	NC_000868	Pyrococcus abyssi GE5	1765118	0.0713
215	NC_003413	Pyrococcus furiosus DSM 3638	1908256	0.3974
216	NC_000961	Pyrococcus horikoshii OT3	1738505	0.0856
217	NC_007348	Ralstonia eutropha JMP134	2726152	0.4505
218	NC_003295	Ralstonia solanacearum GMI1000	3716413	0.9614
219	NC_007761	Rhizobium etli CFN 42	4381608	0.9900
220	NC_007493	Rhodobacter sphaeroides 2.4.1	3188609	0.0892
221	NC_007908	Rhodoferax ferrireducens DSM 15236	4712337	0.7239
222	NC_005027	Rhodopirellula baltica SH 1	7145576	0.5292
223	NC_007925	Rhodopseudomonas palustris BisB18	5513844	0.5676
224	NC_007958	Rhodopseudomonas palustris BisB5	4892717	0.5391
225	NC_005296	Rhodopseudomonas palustris CGA009	5459213	0.4375
226	NC_007778	Rhodopseudomonas palustris HaA2	5331656	0.1193
Continued on next page				

Prokaryote I_r sorted alphabetically, continued from previous page

No.	Accession	Organism	Size	I_r
227	NC_007643	Rhodospirillum rubrum ATCC 11170	4352825	0.6971
228	NC_007940	Rickettsia bellii RML369-C	1522076	0.7991
229	NC_003103	Rickettsia conorii str. Malish 7	1268755	0.0968
230	NC_007109	Rickettsia felis URRWXCal2	1485148	0.9924
231	NC_000963	Rickettsia prowazekii str. Madrid E	1111523	0.0514
232	NC_006142	Rickettsia typhi str. Wilmington	1111496	0.0607
233	NC_007912	Saccharophagus degradans 2-40	5057531	0.4931
234	NC_007677	Salinibacter ruber DSM 13855	3551823	0.1685
235	NC_006905	Salmonella enterica subsp. enterica serovar Choleraesuis str. SC-B67	4755700	0.9917
236	NC_006511	Salmonella enterica subsp. enterica serovar Paratyphi A str. ATCC 9150	4585229	1.1117
237	NC_004631	Salmonella enterica subsp. enterica serovar Typhi Ty2	4791961	1.2962
238	NC_003198	Salmonella enterica subsp. enterica serovar Typhi str. CT18	4809037	1.5869
239	NC_003197	Salmonella typhimurium LT2	4857432	1.0644
240	NC_007954	Shewanella denitrificans OS217	4545906	1.6284
241	NC_004347	Shewanella oneidensis MR-1	4969803	1.4534
242	NC_007613	Shigella boydii Sb227	4519823	1.9191
243	NC_007606	Shigella dysenteriae Sd197	4369232	1.8411
244	NC_004741	Shigella flexneri 2a str. 2457T	4599354	1.6136
245	NC_004337	Shigella flexneri 2a str. 301	4607203	2.1937
246	NC_007384	Shigella sonnei Ss046	4825265	1.7863
247	NC_003911	Silicibacter pomeroyi DSS-3	4109442	1.0099
248	NC_003047	Sinorhizobium meliloti 1021	3654135	1.1582
249	NC_007712	Sodalis glossinidius str. ’morsitans’	4171146	2.6269
250	NC_007622	Staphylococcus aureus RF122	2742531	0.7407
251	NC_002951	Staphylococcus aureus subsp. aureus COL	2809422	0.7696
252	NC_002952	Staphylococcus aureus subsp. aureus MRSA252	2902619	1.3114
253	NC_002953	Staphylococcus aureus subsp. aureus MSSA476	2799802	0.6539
254	NC_003923	Staphylococcus aureus subsp. aureus MW2	2820462	0.6857
255	NC_002758	Staphylococcus aureus subsp. aureus Mu50	2878529	1.3249
256	NC_002745	Staphylococcus aureus subsp. aureus N315	2814816	1.7159
257	NC_007795	Staphylococcus aureus subsp. aureus NCTC 8325	2821361	0.5725
258	NC_007793	Staphylococcus aureus subsp. aureus USA300	2872769	0.6384
259	NC_004461	Staphylococcus epidermidis ATCC 12228	2499279	0.7623
260	NC_002976	Staphylococcus epidermidis RP62A	2616530	1.5083
261	NC_007168	Staphylococcus haemolyticus JCSC1435	2685015	1.3731
262	NC_007350	Staphylococcus saprophyticus subsp. saprophyticus ATCC 15305	2516575	0.8694
263	NC_004116	Streptococcus agalactiae 2603V/R	2160267	1.7840
264	NC_007432	Streptococcus agalactiae A909	2127839	1.7154
265	NC_004368	Streptococcus agalactiae NEM316	2211485	4.8417
266	NC_004350	Streptococcus mutans UA159	2030921	1.5233
267	NC_003098	Streptococcus pneumoniae R6	2038615	1.4171
268	NC_003028	Streptococcus pneumoniae TIGR4	2160837	1.6901
269	NC_002737	Streptococcus pyogenes M1 GAS	1852441	1.1711
270	NC_006086	Streptococcus pyogenes MGAS10394	1899877	1.8149
271	NC_004070	Streptococcus pyogenes MGAS315	1900521	1.7159
272	NC_007297	Streptococcus pyogenes MGAS5005	1838554	1.7275
273	NC_007296	Streptococcus pyogenes MGAS6180	1897573	1.7299
274	NC_003485	Streptococcus pyogenes MGAS8232	1895017	1.8374
275	NC_004606	Streptococcus pyogenes SSI-1	1894275	1.7407
276	NC_006449	Streptococcus thermophilus CNRZ1066	1796226	1.7684
277	NC_006448	Streptococcus thermophilus LMG 18311	1796846	1.6730
278	NC_003155	Streptomyces avermitilis MA-4680	9025608	0.6095
279	NC_003888	Streptomyces coelicolor A3(2)	8667507	1.2834
280	NC_007181	Sulfolobus acidocaldarius DSM 639	2225959	0.0428
281	NC_002754	Sulfolobus solfataricus P2	2992245	1.3393
282	NC_003106	Sulfolobus tokodaii str. 7	2694756	0.3584
283	NC_006177	Symbiobacterium thermophilum IAM 14863	3566135	1.1146

Continued on next page

Prokaryote I_r sorted alphabetically, continued from previous page				
No.	Accession	Organism	Size	I_r
284	NC_006576	Synechococcus elongatus PCC 6301	2696255	0.6855
285	NC_007604	Synechococcus elongatus PCC 7942	2695903	0.6997
286	NC_007516	Synechococcus sp. CC9605	2510659	0.9301
287	NC_007513	Synechococcus sp. CC9902	2234828	0.9351
288	NC_007776	Synechococcus sp. JA-2-3B'a(2-13)	3046682	0.6063
289	NC_007775	Synechococcus sp. JA-3-3Ab	2932766	0.8916
290	NC_005070	Synechococcus sp. WH 8102	2434428	0.6132
291	NC_000911	Synechocystis sp. PCC 6803	3573470	0.7764
292	NC_007759	Syntrophus aciditrophicus SB	3179300	0.7056
293	NC_003869	Thermoanaerobacter tengcongensis MB4	2689445	1.4486
294	NC_007333	Thermobifida fusca YX	3642249	0.6165
295	NC_006624	Thermococcus kodakarensis KOD1	2088737	0.4197
296	NC_002578	Thermoplasma acidophilum DSM 1728	1564906	0.0577
297	NC_002689	Thermoplasma volcanium GSS1	1584804	0.1651
298	NC_004113	Thermosynechococcus elongatus BP-1	2593857	0.9895
299	NC_000853	Thermotoga maritima MSB8	1860725	0.1608
300	NC_005835	Thermus thermophilus HB27	1894877	0.5242
301	NC_006461	Thermus thermophilus HB8	1849742	0.6630
302	NC_007404	Thiobacillus denitrificans ATCC 25259	2909809	0.6284
303	NC_007520	Thiomicrospira crunogena XCL-2	2427734	1.3829
304	NC_007575	Thiomicrospira denitrificans ATCC 33889	2201561	2.6811
305	NC_002967	Treponema denticola ATCC 35405	2843201	0.5407
306	NC_000919	Treponema pallidum subsp. pallidum str. Nichols	1138011	0.9307
307	NC_004551	Tropheryma whipplei TW08/27	925938	1.7737
308	NC_004572	Tropheryma whipplei str. Twist	927303	1.5277
309	NC_002162	Ureaplasma parvum serovar 3 str. ATCC 700970	751719	0.5090
310	NC_002505	Vibrio cholerae O1 biovar eltor str. N16961	2961149	0.9737
311	NC_006840	Vibrio fischeri ES114	2906179	0.9957
312	NC_004603	Vibrio parahaemolyticus RIMD 2210633	3288558	1.1373
313	NC_004459	Vibrio vulnificus CMCP6	3281945	1.0189
314	NC_005139	Vibrio vulnificus YJ016	3354505	0.9815
315	NC_004344	Wigglesworthia glossinidia endosymbiont of Glossina brevipalpis	697724	1.3027
316	NC_002978	Wolbachia endosymbiont of Drosophila melanogaster	1267782	1.6783
317	NC_006833	Wolbachia endosymbiont strain TRS of Brugia malayi	1080084	0.9679
318	NC_005090	Wolinella succinogenes DSM 1740	2110355	1.4649
319	NC_003919	Xanthomonas axonopodis pv. citri str. 306	5175554	0.6897
320	NC_007086	Xanthomonas campestris pv. campestris str. 8004	5148708	0.7958
321	NC_003902	Xanthomonas campestris pv. campestris str. ATCC 33913	5076188	0.9445
322	NC_007508	Xanthomonas campestris pv. vesicatoria str. 85-10	5178466	0.6424
323	NC_006834	Xanthomonas oryzae pv. oryzae KACC10331	4941439	1.5119
324	NC_002488	Xylella fastidiosa 9a5c	2679306	2.7542
325	NC_004556	Xylella fastidiosa Temecula1	2519802	2.2695
326	NC_003143	Yersinia pestis CO92	4653728	1.3248
327	NC_004088	Yersinia pestis KIM	4600755	1.4926
328	NC_005810	Yersinia pestis biovar Medievalis str. 91001	4595065	1.3053
329	NC_006155	Yersinia pseudotuberculosis IP 32953	4744671	0.8542
330	NC_006526	Zymomonas mobilis subsp. mobilis ZM4	2056416	1.1722
